# Embedded Resistance as a Technique to Monitor Concrete Curing

**DOI:** 10.3390/ma17205121

**Published:** 2024-10-21

**Authors:** Etienne Beya Nkongolo, John T. Kevern

**Affiliations:** 1 Civil Engineering, Division of the Natural and Built Environment, School of Science and Engineering, University of Missouri-Kansas City, Kansas City, MO 64110, USA; nkongoloe@umkc.edu; 2 Building Technologies and Science Center, National Renewable Energy Laboratory, Golden, CO 80401, USA

**Keywords:** concrete, curing, membrane forming curing compound, evaluation, moisture loss, embedded resistance

## Abstract

The use of membrane-forming curing compounds on fresh concrete has been widely adopted by many States’ Departments of Transportation as it is feasible where there is a deficiency of water, on sloping surfaces where curing with water is challenging, and in cases where large areas like pavement have to be cured. However, the evaluation of the curing compound application effectiveness is difficult because most of the evaluation test methods are not performed during the early age of the concrete. Moreover, the ASTM C156 standards test of water retention for the qualification of curing compounds has met criticism as the moisture retention is performed only on the mortar specimens, with a fixed application rate and curing condition. Therefore, in this study, the embedded resistance technique was used as a test replacement for the moisture retention test to assess concrete curing. The findings from this study showed that a correlation can be found between the moisture retention test and the embedded resistance test. Based on the findings, the embedded resistance test could be a suitable replacement for the moisture loss test, because the test is much simpler and quicker to be performed both in the lab and in the field.

## 1. Introduction

The curing of concrete and especially concrete pavement has significant effects on the long-term performance and durability due to the high amount of exposed surface which is also the functional surface. The application of a membrane forming curing compound (MFCC) is a relatively low-cost technique to reduce moisture loss from the concrete pavement and ultimately improving the quality of concrete pavement surface [1]. Especially for large installations where wet curing or covering with plastic is impractical and expensive, MFCC is the only viable option [2]. MFCCs are solutions of wax or resin in water or other chemical solvents. When applied to the surface of fresh concrete, the solution breaks down and the water or solvent evaporates leaving behind the wax or resin to form a membrane which minimizes evaporation and supports continued hydration [3,4]. According to ASTM C309, MFCCs are classified into types based on color, solid constituents, and solids chemistry [5]. Moreover, MFCCs are also tested for compliance with moisture retention, coverage, drying time, flash point, and reflectance [1]. ASTM C309 [5] specifies a maximum moisture loss limit of 0.55 kg/m^2^ as tested according to ASTM C156, while ASTM C1315 [6] restricts the maximum moisture loss to no more than 0.4 kg/m^2^ within 72 h [7]. In the United States (U.S.), there is not a single specification for MFCC with many including 24 h. moisture loss requirements, limiting the time of application, or requiring multiple applications. No technique currently exists to assess the application rate in the field with the visual comparison to a sheet of printer paper common, but not quantifiable or enforceable. The large surface area-to-volume ratio of concrete pavement is a challenge for maintaining uniformity across the pavement [8,9].

Most specifications for MFCC application require an application rate of 3.7–5 m^2^/L with little regard for field conditions or the potential for over- or under-curing [7]. Other than ASTM C309 and the previously mentioned adjustments to moisture loss, no current specifications provide guidance on how to qualify curing effectiveness in terms of the type of performance expected for given environmental conditions. Unfortunately, curing effectiveness is not a singular value such as strength and is attached to a variety of concrete performance indicators, including drying shrinkage, cracking, scaling, joint spalling, curling and warping, and freeze–thaw deterioration, amongst many others. For instance, concrete plastic shrinkage occurs when the rate of water loss from the surface exceeds the rate at which bleed water is available and is directly linked to curing effectiveness [8]. The evaporation rate is a function of concrete temperature, air temperature, wind speed, relative humidity, color of the fresh concrete, and solar gain [10]. However, ASTM C156 [7] has met criticism as the moisture retention is performed only on the mortar specimens with a fixed application rate and curing condition and has low precision with a single-operator standard deviation of 0.13 kg/m^2^ and a multi-laboratory deviation of 0.30 kg/m^2^ [10,11,12]. While the usefulness of a laboratory-based quality control test is arguable, moisture loss using any actual adapted measure is not possible in the field.

Besides bulk moisture loss, the effectiveness of a curing compound is also governed by the integrity of the membrane formed. Factors that impact the integrity of the membrane include the effectiveness of the curing compound material [13,14], the amount applied, the application timing, the surface texture [1], concrete bleeding [15], application uniformity, and environmental conditions [16]. The American Concrete Institute (ACI) recommends the optimal time to apply is after the final surface finishing, when the bleed water has evaporated from the concrete surface [17]. However, this guidance is complicated by evaporative conditions that, when too high, can prematurely signal the end of bleeding or, when too low, extend the period that water appears on the surface. The former condition has been particularly troublesome, as multiple studies have noted that pinholes and cracks can form in the membrane as bleed water can segregate the freshly placed curing compound [1,12]; meanwhile, the latter can create plastic shrinkage cracking.

Concrete goes through a phase change from a plastic phase to a solid at an early age. This transition is mainly caused by the hydration reaction [18]. During the early age of concrete, cement reacts with water to produce hydration reaction products, and the excess water evaporates due to drying, leading to concrete microstructure development. However, at an early age, the presence of moisture in concrete makes concrete less resistant to current flow, as the electrical current in concrete moves through the pore spaces. With time, more pore spaces in concrete are filled up with hydration reaction products. Subsequently, concrete becomes more resistant to the current flow, as the electrical conductivity ability of concrete depends on the conductivity of the fluids inside the interconnection of the pore system, the degree of saturation of the concrete, and the permeability. Therefore, using the resistance technique to trace moisture content in concrete can be an important tool to assess curing compound application effectiveness on fresh concrete.

In this study, we investigated the effects of curing compound application time on freshly placed concrete, the effect of curing conditions as well on the performance of concrete cured before and after the initial setting time, and lastly the effect of curing compound application rates and uniformity during the application. Tests were indexed against the standard moisture loss testing in addition to a newly developed real-time assessment of moisture loss and curing by embedded resistance. Moisture is critical for developing desirable concrete properties but not easily measured in practice and especially at early ages. This study is significant because embedded resistance can provide a correlated measure of moisture loss from field measurements.

## 2. Materials and Methods

In this study, a conventional highway paving mixture was used, as shown in Table 1, which contains Type IL cement (Central Plains, Kansas City, MO, USA), river sand (Holliday Sand, Kansas City, MO, USA), and optimized gradation of limestone (Martin Marietta, Kansas City, MO, USA) with a 25 mm nominal maximum aggregate size. The MFCC was a poly alpha methyl styrene (PAMS) (2200 White, WR Meadows, Hampshire, IL, USA) meeting ASTM C309 [5] Type 2B requirements.

### Mixing and Testing

Concrete was produced using a rotating drum-type mixer following ASTM C192 [19]. The mixture contained a vinsol resin-based air-entraining admixture (AirAvalon, GCP Applied Technologies, Alpharetta, GA, USA) and polycarboxylate water reducer (ADVA190, GCP Applied Technologies, Alpharetta, GA, USA). Aggregate quality control was maintained by washing coarse aggregate, oven drying both fine and coarse aggregate 24 h prior to mixing and allowing it to cool before mixing. Baseline characterization testing included air content test following ASTM C231 [20], slump ASTM C143 [21], unit weight ASTM C138 [22], compressive strength ASTM C39 [23,24], surface resistivity AASHTO T358 [25], and setting time using Vicat apparatus (ASTM C-191) [26] and ultrasonic pulse velocity (UPV) [27].

**Figure 1:**
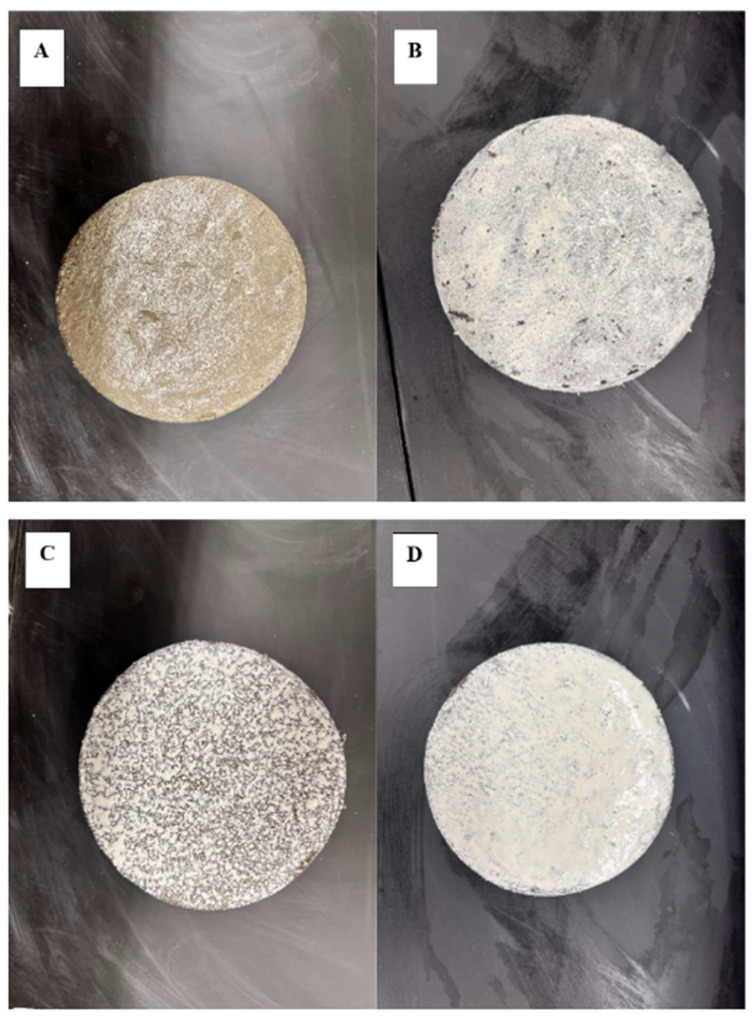
Concrete application rates: (**A**) uncured; (**B**) 9.8 U; (**C**) 4.4 NU; (**D**) 4.4 U.

For the moisture retention and embedded resistance tests, the investigation was performed using four evaporation rates and four application rates as shown in Table 2. For the remainder of the article, the evaporative rates are identified as low (10 °C, 75% RH), medium (23 °C, 50% RH), moderate (32 °C, 50% RH), and high (38 °C, 32% RH). The effect of uniformity was determined using four application rates: uncured (0 m^2^/L), 9.8 m^2^/L uniform (9.8 U), 4.4 m^2^/L non-uniform (4.4 NU), and 4.4 m^2^/L uniform (4.4 U). Figure 1 shows an example of each. Specimens were cured using a pump sprayer; for the 9.8 samples, a brush was used.

A moisture loss test was performed on both the specified mortar and the actual concrete to assess anticipated differences in the field according to the ASTM C156 [7] process. The concrete mass was recorded at the ages of 0,24, 48, and 72 h, and the mass loss was determined following the equation provided by the ASTM C156. Additionally, for the concrete moisture retention test, the following three additional variables were used: application time, application rate, and evaporative rate. The effect of application time was investigated by applying MFCC immediately after the final surface finishing (approximately 30 min after mixing and 120 min before the initial set) and after the bleed water was no longer present (after the initial set).

**Figure 2:**
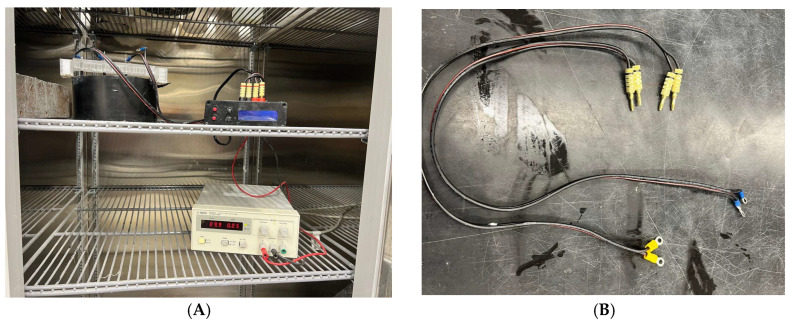
Embedded resistance set-up. (**A**) Data logger resistance probes connected to concrete. (**B**) Resistance probe.

The embedded resistance test was performed on concrete cast in 150 mm × 75 mm cylinder molds. The variables investigated were only application rates, evaporative rates (as presented in Table 2), and the probe depths. After applying the curing compound, the resistance probes were embedded to record the resistance at the surface of the concrete just below the curing compound layer and at 12.5 mm into the concrete, below the cure-affected depth. The embedded resistance probe used a two-pin arrangement in a non-conductive plastic frame to hold four stainless steel probes, two for each depth. The sides of the probes were protected using nylon sleeves to ensure that data were collected only from the concrete at the probe tip location. The frame was set 25 mm above the surface to allow the free movement of air at the concrete surface A data logger was used to record resistance development with time at 5 min intervals. The complete set-up is shown in Figure 2. Resistance was recorded at 5 min increments until the maximum cutoff value of 160 kΩ/cm was reached.

## 3. Results and Discussion

### 3.1. Concrete Baseline Properties

The baseline concrete properties are shown in Table 3. All results are acceptable and expected for paving concrete suitable for application in freeze–thaw climates. The average 28-day compressive strength was 41 Mpa after lime water curing. Surface resistivity was 15 kΩ-cm, classified as moderate chloride ion permeability by AASHTO T277 or ASTM C1202 [28,29].

### 3.2. Moisture Retention Test

**Figure 3:**
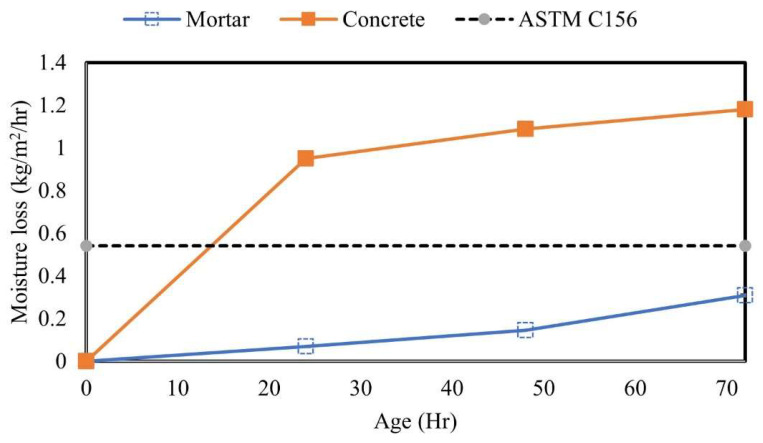
Comparison between ASTM C156 and concrete specimens cured at 4.4 m^2^/L in 38C32%.

Figure 3 shows the comparison of moisture loss performed on the standard reference mortar and on the paving concrete cured following the ASTM C156 standard. As expected, both specimens experienced moisture loss throughout the curing period, with the most moisture loss observed during the first 24 h. After 72 h of curing, the mortar specimens had less moisture loss compared to concrete. The high moisture loss observed in concrete could be due to the presence of coarse aggregates that cause more bleed water during the early time stages of concrete.

**Figure 4:**
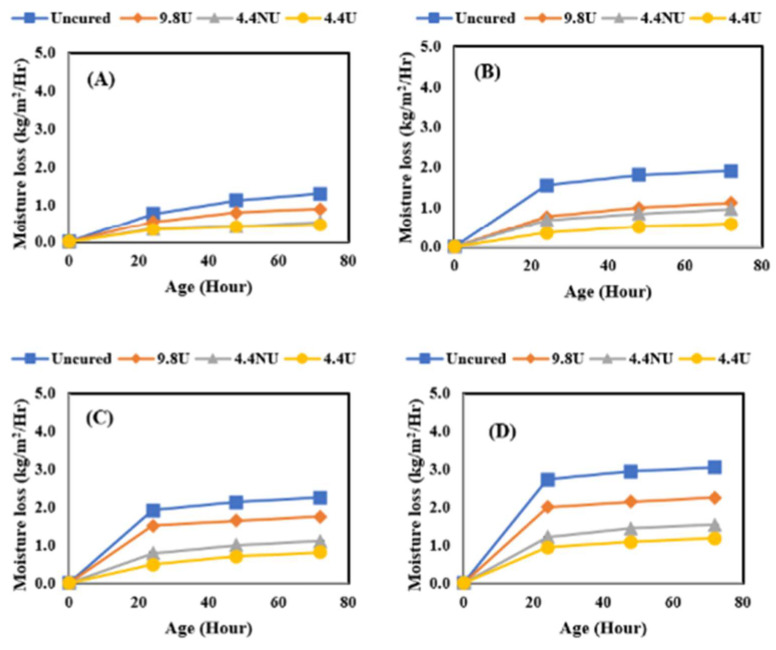
The effect of evaporative conditions on moisture loss. (**A**) Low; (**B**) medium; (**C**) moderate; (**D**) high evaporative conditions.

Figure 4 presents the comparison of moisture loss results performed on concrete only while varying the application rates and curing conditions. The four application rates were 0 m^2^/L, 9.8 m^2^/L applied using a brush, 4.4 m^2^/L non-uniform applied using a pump sprayer, and lastly, 4.4 m^2^/L uniformly applied using a pump sprayer; the four evaporative conditions were identified as low, medium, moderate, and high. The low evaporative condition (A) was performed at 10 °C and 75% RH with an estimated evaporation rate of 0.00732 kg/m^2^/h. The medium evaporative condition (B) was performed at 23 °C and 50% RH with an evaporation rate of 0.1515 kg/m^2^/h. The moderate evaporative condition (C) was performed at 32 °C and 50% RH with an evaporation rate of 0.4098 kg/m^2^/h. The high evaporative condition (D) was performed at 38 °C and 32% RH with an evaporation rate of 0.7585 kg/m^2^/h. The data presented are average values from triplicate testing. As expected, the moisture loss from the samples increased with higher evaporative conditions with the uncured samples having the greatest moisture loss. The effect of application rate and quality became more noticeable with the increased evaporation rate. At the lowest two evaporation rates, the uniformity and rate were not significant factors. At the highest two evaporation rates, the uniformity was less important than the application rate.

**Figure 5:**
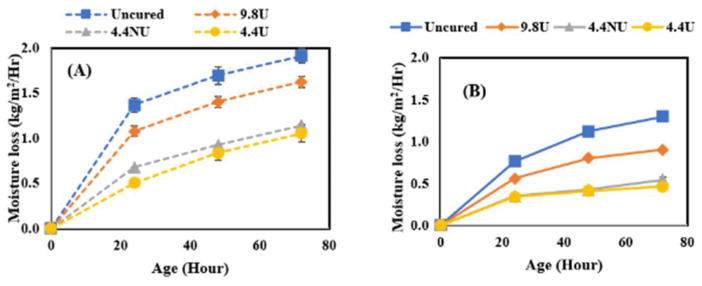
Low evaporative condition moisture loss results. (**A**) Before set results; (**B**) after set results.

**Figure 6:**
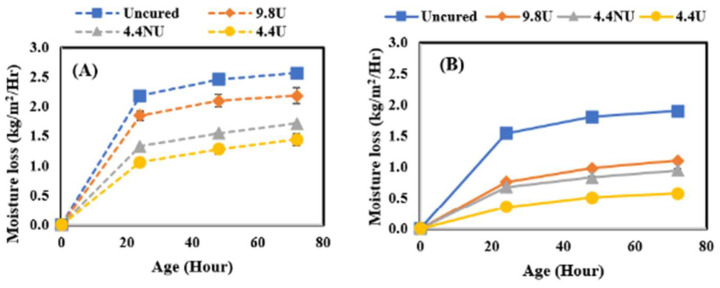
Medium evaporative condition moisture loss results. (**A**) Before set results; (**B**) after set results.

**Figure 7:**
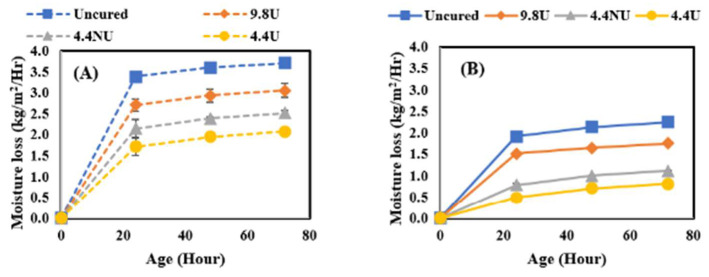
Moderate evaporative condition moisture loss results. (**A**) Before set results; (**B**) after set results.

**Figure 8:**
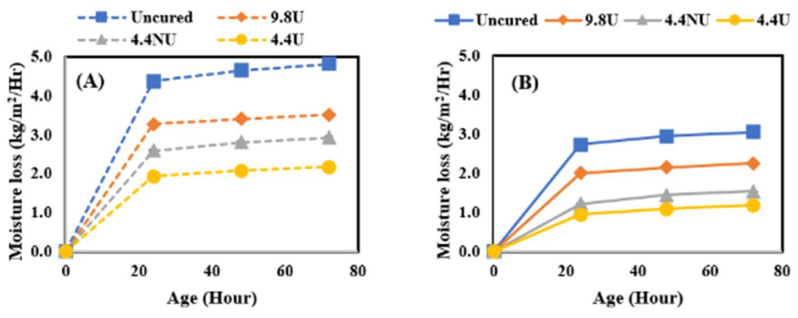
High evaporative condition moisture loss results. (**A**) Before set results; (**B**) after set results.

In this study, the effect of curing compound application time was further investigated by comparing the moisture loss behavior of concrete when MFCC was applied directly after final surface finishing (120 min before the initial set) or after the initial set (210–240 min from the time when cement met water). It should be noted that the evaporative conditions of the laboratory are quite low resulting in a much longer time for bleed water to evaporate than would be experienced in field conditions. The comparison was performed by comparing moisture loss after 72 h of curing for all four application rates identified as uncured, 400 U, 180 NU, and 180 U and four curing conditions. Figure 5 shows the results for the lowest, Figure 6 for the medium, Figure 7 for the moderate, and Figure 8 for the highest evaporative conditions. In all cases, the samples where MFCC was applied while bleed water was present had the greatest moisture loss. While some of the difference can be attributed to evaporation during the 120 min between the before and after conditions, the additional moisture loss is more than can be attributed to evaporation alone. It has been well described that in all but the harshest conditions, most of the bleed water is reabsorbed as pores empty during hydration [30]. Applying the MFCC to wet concrete likely dilutes the chemicals and creates a more permeable final film. Whatever the mechanism, it is important to ensure that the MFCC is applied after bleed water is no longer present. While the timing is important, the slow loss of bleed water corresponds to low evaporative conditions which are much less sensitive to curing effects and likely not of high importance.

Additionally, statical t-tests were performed between the application rates for both sets of curing application times and curing conditions. The t-test demonstrated the difference between the application rates at a 95 percent confidence level. The major highlight was that there was no significant difference between 4.4 NU and 4.4 U for the sample cured at lower evaporative conditions and the finding simply means that at lower evaporative conditions, the application quality is not important. The best option is to apply the correct amount of MFCC on the concrete without overly focusing on uniformity. Additionally, the effect of the application time was significant, with the initial set of specimens presenting high moisture loss even though a curing compound was applied to prevent moisture from evaporating. This indicates that curing concrete too soon does not benefit the performance of concrete in terms of moisture retention capacity; therefore, the concrete curing time is an important factor to consider in order to produce good concrete.

### 3.3. Embedded Resistance Data

**Figure 9:**
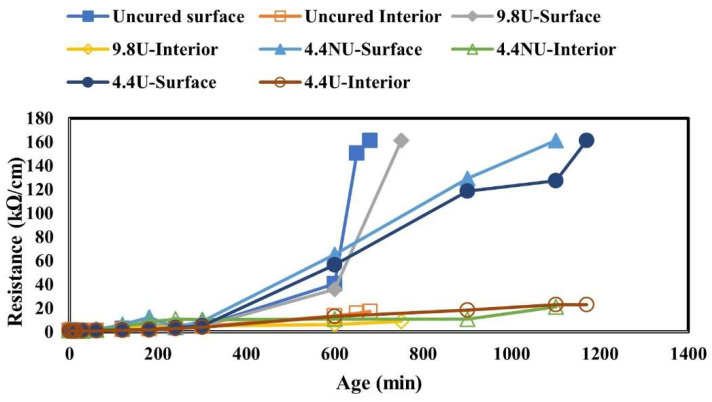
Concrete drying behavior investigated using resistance techniques.

The embedded resistance results for moderate evaporative condition (32C50%RH) for all the application rates, uncured, 9.8 U, 4.4 NU, and 4.4 U, are shown in Figure 9. The surface resistance data increased with time at different rates depending on the application rates and application quality. During the early age life of concrete, concrete transitions from the plastic phase to the solid phase. The uncured specimens were first to reach the maximum cutoff point of 160 kΩ/cm, followed by 9.8 U and 4.4 NU, and lastly, the 4.4 U was the last to reach the maximum resistance. Additionally, the resistance values at the 12.5 mm depth were similar for all the application rates, demonstrating that 12.5 mm is below the cure-affected zone.

The concrete surface is cured to prevent or slow moisture from evaporating. Concrete during plastic states/phase is less resistant to current flow due to the presence of moisture or fluid in the pore’s spaces. However, with time, the moisture/water in the concrete is used up through a hydration reaction, and some evaporate due to the drying caused by the ambient conditions, thus causing the concrete to become more resistant to current flow. A curing compound is applied to slow moisture loss and favor hydration. The effect of the application rates was investigated by comparing time to maximum resistance for all the evaporation rates used. The results revealed that the uncured specimens were faster to reach the maximum resistance, followed by 9.8 U, 4.4 NU, and lastly, 4.4 U, and thus indicating the effect of application rates on the moisture retention behavior of concrete.

**Figure 10:**
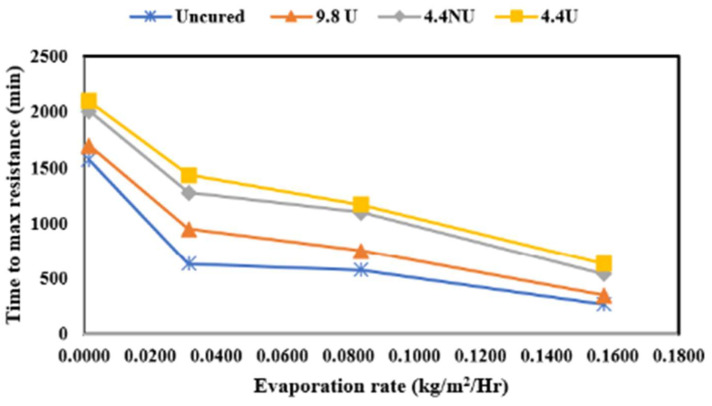
Time to maximum surface resistance for all the application rates and evaporation rates used in this study.

The effect of the evaporative conditions was investigated by curing concrete in four evaporation rates named low (0.00732 kg/m^2^/h), medium (0.1515 kg/m^2^/h), moderate (0.4098 kg/m^2^/h), and high (0.7585 kg/m^2^/h). For each evaporation rate, concrete specimens were cured using the four application rates of uncured, 9.8 U, 4.4 NU, and 4.4 U. Presented in Figure 10 is the surface resistance time to maximum resistance for each application rate plotted against the evaporation rate.

The results revealed that time to maximum surface resistance depended on the application rate and curing conditions. Resistance increases in all the cases (all the application rates and curing conditions) due to the concrete drying process and hydration reaction. However, the rates of resistance increase depended on the curing conditions (application and evaporative rates). Specimens cured at higher evaporative rates presented a faster resistance increase than the rest of the conditions, and the low evaporative rates specimens presented a slower resistance increase. Based on the findings from this study, it can be said that the resistance increase is inversely proportional to the evaporative rates. Increasing the evaporative condition decreases the time to the maximum resistance cut point, and the observation was the same for all of the curing conditions.

### 3.4. Correlation between Moisture Loss and Embedded Resistance Data

**Figure 11:**
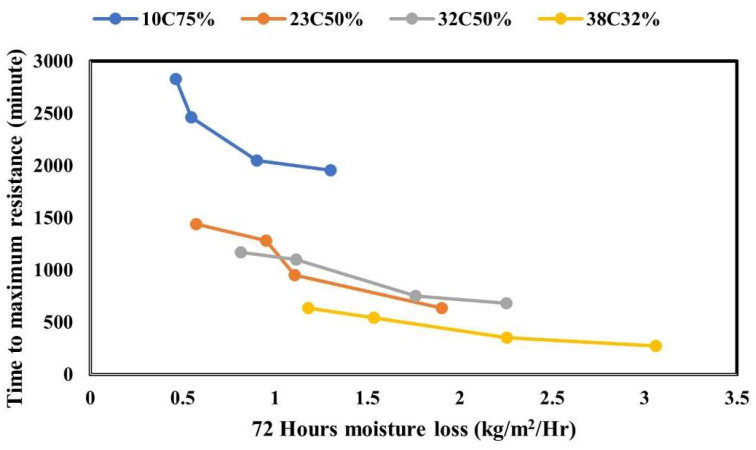
Relationship between moisture loss and embedded resistance.

The relationship between the moisture loss and embedded resistance was developed by plotting the surface resistance time to the maximum resistance value against moisture loss after 72 h of curing. The results presented in Figure 11 are for all four application rates (uncured, 9.8 U, 4.4 NU, and 4.4 U) and the four evaporative conditions mainly low (0.00732 kg/m^2^/h), medium (0.1515 kg/m^2^/h), moderate (0.4098 kg/m^2^/h), and high (0.7585 kg/m^2^/h). The embedded resistance test is inversely proportional to the moisture loss. The results demonstrated that specimens with higher moisture loss have a shorter time to the maximum resistance value. In all cases, regardless of the evaporative conditions, uncured specimens had a higher moisture loss than the other specimens and at the same time presented a faster surface resistance, followed by 9.8 U and 4.4 NU, lastly, the 4.4 U specimens had a low moisture loss in all the evaporative conditions. This trend was consistent with the embedded resistance results where the 4.4 U specimens had the longest time to the maximum resistance values.

## 4. Conclusions

The research presented in this paper investigated the effect or impact of the membrane-forming curing compound on concrete pavement by varying time to curing, application rates, and evaporative conditions. The findings from this study demonstrated the following:

1. Curing concrete early while bleed water is still available affects the MFCC performance and causes more moisture loss.
2. Concrete should be cured at all costs, as in all cases, uncured specimens have a higher moisture loss than the cured specimens, regardless of the application rates.
3. Additionally, at lower evaporative conditions, curing compound application quality does not really affect concrete performance; the effect of curing quality is more significant at higher evaporative conditions than at lower evaporative conditions.
4. The effect of the application rates and evaporative conditions was also proven to be significant while using the embedded resistance test.

Furthermore, a correlation between the moisture loss test and embedded resistance test was developed, demonstrating that embedded resistance can be used to assess concrete moisture loss both in the laboratory and in the field because the test is a much simpler and quicker test to perform and could be a suitable replacement for moisture loss test. Future research should include indexing the resistance and moisture loss to properties of concern such as the degree of hydration and microstructural porosity.

## Figures and Tables

**Table 1:** Lab testing mixing design.

Materials	Mix Proportions, kg/m^3^
Cement 1 L (12%)	325
Fine agg.	795
Intermediate agg.	695
Coarse agg.	385
Water	130
w/cm	0.42

**Table 2:** Testing curing conditions.

Curing Conditions			Term	
Temperature (°C)	Relative	Evaporation Rates (kg/m^2^/h)		Application Rates (m^2^/L)
	Humd. (%)			
38	32	0.76	Low	0 (Uncured)
32	50	0.41	Medium	9.8 U
23	50	0.15	Moderate	4.4 NU
10	75	0.01	High	4.4 U

**Table 3:** Baseline properties results.

Slump (mm)	Unit Weight, kg/m^3^	Air Content (%)	Initial Set (min)	Final Set (min)
37.5	2403	5.2	155	240

## Data Availability

The original contributions presented in the study are included in the article, further inquiries can be directed to the corresponding author.
